# Can We Trust PAICs in Rare Diseases? Methodological Challenges and Limitations

**DOI:** 10.3390/jmahp14010014

**Published:** 2026-03-06

**Authors:** Mikolaj Parkitny, Samuel Aballéa, Piotr Wojciechowski, Mondher Toumi

**Affiliations:** 1 Public Health Department, Faculty of Medicine, Aix-Marseille University, 13385 Marseille, France; 2 Clever-Access, Wadowicka 8a, 30-415 Krakow, Poland; 3 InovIntell, 215 rue du Faubourg St Honoré, 75008 Paris, France

**Keywords:** matching-adjusted indirect comparison, simulated treatment comparison, rare diseases

## Abstract

Population-adjusted indirect comparisons (PAICs), including Matching-Adjusted Indirect Comparison and Simulated Treatment Comparison, are increasingly used to inform health technology assessments. These methods offer a pragmatic approach to generating comparative evidence between treatments when head-to-head trial data are unavailable and standard indirect treatment comparison methods are unfeasible. In rare diseases, however, PAICs often face substantial methodological challenges arising from small sample sizes, limited covariate overlap, and the frequent use of unanchored comparisons that rely on unverifiable assumptions. These limitations can lead to unstable estimates, reduced precision, and bias that may undermine the reliability of findings. Methodological refinements—such as optimized weighting, Bayesian approaches, and doubly robust estimators—provide some improvements but do not resolve these fundamental issues. Current European Joint Clinical Assessment guidance recommends that anchored PAICs be applied with great caution, while unanchored PAICs are considered *highly problematic*, and other methods *should be used instead*. We argue that PAICs can play a supportive role within a multidimensional and deliberative HTA process, contributing to comparative assessment alongside other evidence sources when available data are limited. However, their results require careful interpretation and transparent communication of uncertainty. Future research should prioritize the further development of formal frameworks to quantify bias and systematically assess robustness, thereby preventing overstatement of the credibility of PAIC-derived evidence in rare disease contexts.

## 1. Introduction

Comparative evaluation of treatment effectiveness is a central component of health technology assessments (HTAs), which guide regulatory approval and reimbursement decisions by agencies such as the National Institute for Health and Care Excellence (NICE). While randomized controlled trials (RCTs) are considered the gold standard for generating comparative evidence, head-to-head RCTs between new treatments and existing alternatives are often unavailable, especially in rare diseases [1]. In such cases, indirect treatment comparisons (ITCs) are commonly employed to estimate relative treatment effects and support HTA decision-making [2].

Conventional ITCs, such as Bucher’s ITC or network meta-analysis (NMA), estimate relative treatment effects between interventions assessed in RCTs which share a common comparator and involve sufficiently similar populations with respect to effect-modifying factors [2,3]. When these assumptions are violated, population-adjusted indirect comparisons (PAICs) may serve as a methodological alternative [4]. These methods require individual patient data (IPD) for one trial (typically from the submitting company) and aggregate-level data (AgD) for the relevant comparator trial [5]. PAICs adjust for cross-trial differences in baseline characteristics prior to conducting the comparison, thereby facilitating a more accurate estimation of relative treatment effects [5].

The two most widely used PAIC methods include Matching-Adjusted Indirect Comparison (MAIC) and Simulated Treatment Comparison (STC), with MAIC being the more commonly applied approach [6,7,8]. Both methods are specifically designed for two-study indirect comparisons and can be applied in both connected and disconnected evidence networks, although the latter require stronger assumptions. As such, they are particularly valuable when at least one treatment of interest has been evaluated in single-arm study, where conventional ITC methods are not applicable [6,9,10]. In MAIC, individual patients in the IPD are assigned weights so that the weighted summary statistics of baseline covariates match the reported aggregate characteristics of the comparator trial. The relative treatment effect is then estimated by comparing the reweighted treatment effect from the IPD population with the published treatment effect from the comparator trial [9]. STC, by contrast, fits a regression model to the IPD to estimate the relationship between covariates and outcomes. This model is subsequently used to predict treatment outcomes in the comparator population based on its reported covariate profile [10].

PAICs have become increasingly common in rare disease evaluations due to the distinctive challenges associated with this therapeutic area [11]. Small patient populations frequently require more restrictive inclusion criteria, increasing the likelihood of between-trial differences in study populations. Additionally, treatments for rare diseases—often designated as orphan drugs—are commonly investigated in single-arm trials, driven by limited patient availability, ethical considerations, and logistical constraints [12].

While PAICs offer a pragmatic and often necessary approach in data-limited settings, their application in rare diseases raises substantial methodological concerns. In this paper, we outline these challenges and argue that they are not merely technical limitations but may fundamentally undermine the validity and reliability of PAIC-derived results. As such analyses increasingly inform access and reimbursement decisions, it is crucial to recognize their limitations and to develop methodological innovations that both enhance the credibility of these comparisons and enable systematic assessment of their robustness.

## 2. MAIC and STC Methodology

MAIC and STC can be implemented in either anchored or unanchored forms. In unanchored analyses, there is no common comparator between trials, which are often single-arm studies. In this setting, the absolute effects observed in the respective treatment arms are compared directly. Consequently, adjustment must account for both prognostic variables—patient characteristics that influence outcomes irrespective of treatment—and effect modifiers, which alter the relative effect of one treatment compared with another.

In contrast, an anchored comparison is conducted when both treatments of interest are evaluated relative to a common comparator, typically placebo or standard of care. In this case, adjustment is required only for effect modifiers, as the impact of prognostic factors cancels out within each randomized trial.

The following methodology focuses on MAIC and STC in the unanchored setting, which is common in PAICs in rare diseases. Specifically, we consider the estimation of the treatment effect of Treatment A versus Treatment B, where IPD are available from a single-arm study of Treatment A (the A study), and only aggregate-level data are available from a separate single-arm study of Treatment B (the B study). The summary of methodological aspects of MAIC and STC may be found in Table 1.

### 2.1. MAIC

MAIC assigns weights to individuals in Study A to achieve balance in covariates between the populations of Studies A and B. The weights are derived from a propensity score model:

(1) $$\ln\left(w_{i}\right)=\alpha_{0}+X_{i}\alpha_{1},$$

where $\alpha_{0},$ $\alpha_{1}$ are the parameters, and $X_{i}$ represents the covariates for individual i. The weight $w_{i}$ reflects the odds that individual i would be selected into Study B rather than Study A, based on observed characteristics.

Because IPD are not available for Study B, standard maximum likelihood estimation of the parameters is not feasible. Instead, the ${\hat{\alpha }}_{1}$ is typically estimated using the method of moments, ensuring that the weighted means of selected covariates in Study A match the corresponding published aggregate values from Study B (${\bar{X}}_{B}$), i.e., [9]:

(2) $$\frac{\sum_{i=1}^{N_{A}}w_{i}X_{i}}{\sum_{i=1}^{N_{A}}w_{i}}=\frac{\sum_{i=1}^{N_{A}}exp(X_{i}{\hat{\alpha }}_{1})X_{i}}{\sum_{i=1}^{N_{A}}exp(X_{i}{\hat{\alpha }}_{1})}={\bar{X}}_{B}.$$

The intercept term ($\alpha_{0}$) is omitted as it is common to all weights and cancels out. Consequently, the weights are identified only up to a multiplicative scalar.

Next, the covariates included in X are centered on the published summary values for the comparator baseline characteristics (${X_{i}^{*}=X_{i}-\bar{X}}_{B}$) and, thus, the estimation problem then reduces to solving:

(3) $$\frac{\sum_{i=1}^{N_{A}}exp(X_{i}^{*}{\hat{\alpha }}_{1})X_{i}^{*}}{\sum_{i=1}^{N_{A}}exp(X_{i}^{*}{\hat{\alpha }}_{1})}=0.$$

The weights are estimated by minimizing the objective function:

(4) $$Q\left({\hat{\alpha }}_{1}\right)=\;\sum_{i=1}^{N_{A}}exp(X_{i}^{*}{\hat{\alpha }}_{1}).$$

Since the derivative of $Q\left({\hat{\alpha }}_{1}\right)$ with respect to ${\hat{\alpha }}_{1}$ is the numerator in (3), its minimization yields the same solution as directly enforcing the moment conditions.

Given the weights $w_{i}$, the adjusted mean outcome for treatment A in the target population of Study B (${\hat{y}}_{A}^{B}$) is computed as the weighted average of the observed outcomes in Study A:

(5) $${\hat{y}}_{A}^{B}\;=\frac{\Sigma w_{i}y_{i}^{A}}{\Sigma w_{i}}.$$

Notably, at this stage, implicit assumptions are made about the relationship between outcomes and covariates, implying the existence of an underlying outcome model although it is not explicitly estimated.

The standard error of the adjusted treatment effect is typically estimated using either a robust sandwich variance estimator or non-parametric bootstrapping.

To estimate the treatment effect of A versus B in the Study B population, Bucher’s indirect comparison method is applied on the linear predictor scale [3]:

(6) $${\hat{d}}_{AB}^{B}=g\left({\hat{y}}_{A}^{B}\right)-g\left({\hat{y}}_{B}^{B}\right),$$

where g(⋅) is a suitable link function (e.g., the logit function for binary outcomes), and ${\hat{y}}_{B}^{B}$ represents the reported aggregate outcome for treatment B in its original population.

As MAIC is a reweighting technique, it reduces the effective sample size (*ESS*) of the adjusted IPD dataset. The approximate *ESS* is given by:

(7) $$ESS\;\approx \frac{{\left(\Sigma w_{i}\right)}^{2}}{\Sigma {w_{i}}^{2}}.$$

A significant reduction in *ESS* indicates limited overlap in covariate distributions between Studies A and B, which may compromise the reliability and precision of the adjusted treatment effect estimate.

### 2.2. STC

In unanchored STC, a regression model is fitted to IPD from Study A to estimate the relationship between baseline covariates and outcomes under treatment A. The model is specified as follows:

(8) $$g(\theta_{i}^{A}(X_{i}))\;=\;\beta_{0}\;+\;\beta_{1}^{T}X_{i},$$

where $\theta_{i}^{A}(X_{i})$ is the expected outcome for individual i with covariates $X_{i}$ in the A population. The function g(·) represents an appropriate link function (e.g., the logit function for binary outcomes), $\beta_{0}$ is the intercept, and $\beta_{1}$ is a vector of coefficients capturing the effects of prognostic variables and effect modifiers.

In conventional STC, once the model parameters are estimated, mean values of the baseline covariates from the comparator study (Study B) are substituted into the fitted model to predict the outcome for treatment A in the Study B population:

(9) $${\hat{d}}_{A}^{B}\;=\;g\left({\hat{\theta }}_{A}^{B}\right)=g\left(\;{\hat{\beta }}^{0}+\;{\hat{\beta }}_{1}^{T}{\bar{X}}_{B}\right),$$

where ${\bar{X}}_{B}$ denotes the vector of mean baseline characteristics reported for Study B. This substitution procedure yields a conditional estimate of the outcome, corresponding to an “average” patient in the comparator population.

Although conventional STC is referenced in methodological guidelines [13], it is associated with several limitations. For non-linear outcome models, aggregation bias may arise [4,14]. Moreover, conventional STC yields the conditional estimates of non-collapsible effect sizes—such as log odds ratio or log hazard ratio—that are not compatible with the marginal effects from comparator study, leading to biased indirect comparisons [15].

To address this limitation, STC with G-computation has been proposed [16]. This approach produces marginal estimates of treatment outcomes, reflecting the average expected outcome across the full covariate distribution of the comparator population. These marginal estimates are more appropriate for population-level decision-making and align better with the evidentiary requirements of HTA bodies.

In STC with G-computation, instead of substituting mean covariate values, the model predictions are integrated over the full covariate distribution from Study B:

(10) $${\hat{d}}_{A}^{B}\;=g\left(\int g^{-1}\left({\hat{\beta }}^{0}+\;{\hat{\beta }}_{1}^{T}X_{j}^{B}\right)f_{B}(X)dX\right)$$

where $f_{B\left(X\right)}$ represents the covariate distribution in Study B. In practice, the integral is approximated via Monte Carlo, by averaging predictions across a sample of individuals $X_{j}^{B}$ drawn from the comparator population. Because only summary statistics are available for Study B, $X_{j}^{B}$ is often simulated using a Gaussian copula whose marginals reproduce the reported Study B summaries (means/SDs or proportions). Notably, reconstructing the comparator covariate distribution using parametric assumptions such as Gaussian copulas requires specification of marginal distributions and correlation structures. These quantities are often unreported in aggregate data, necessitating additional assumptions and thereby increasing uncertainty, with the potential to introduce bias into the resulting estimates.

As with MAIC, the final population-adjusted treatment effect for A versus B in the Study B population is estimated using Bucher’s indirect comparison method on the linear predictor scale. In the following sections, the term STC will refer specifically to Simulated Treatment Comparison implemented using the G-computation framework.

## 3. Methodological Challenges for PAICs in Rare Diseases

While PAICs offer a pragmatic alternative in the absence of head-to-head trials—and appear particularly well suited to rare disease research—their validity is frequently undermined by a set of interrelated methodological challenges.

First, pivotal trials assessing orphan drugs typically enroll far fewer participants than trials for more prevalent conditions. For example, the median number of patients enrolled in cancer trials supporting U.S. FDA approvals was 199 for rare orphan indications, 85 for ultra-rare orphan indications, and 521 for non-orphan indications [17].

Second, rare diseases are highly heterogeneous, exhibiting wide variation in prevalence, rates of progression, and degrees of clinical heterogeneity [18,19,20]. These can influence both symptom presentation and disease course [19]. Moreover, knowledge of the natural history and pathophysiology of many rare diseases remains limited. To minimize variability, clinical trials often apply narrow eligibility criteria. Consequently, the study populations may differ substantially with respect to prognostic factors or effect modifiers. This between-trial heterogeneity increases the risk of poor covariate overlap across studies used in the ITC.

Third, the evidence for rare diseases often relies on single-arm trials. Among cancer drug approvals, 44% of pivotal trials for rare orphan indications and 85% for ultra-rare indications were single-arm studies, compared with 21% for non-orphan indications [17]. This dependence on single-arm evidence often necessitates unanchored PAICs, which are inherently more methodologically demanding due to their reliance on stronger assumptions and higher sensitivity to model misspecification.

To illustrate the methodological implications of these challenges, we consider a common scenario encountered in rare disease contexts: (1) limited IPD sample sizes (100–200 patients), (2) poor covariate overlap between studies, and (3) the need for unanchored comparison. Far from being exceptional, this scenario is representative of the typical setting in which PAICs are applied [6].

We begin by discussing the implications of limited sample size and poor covariate overlap, which are closely interrelated and frequently co-occur in rare disease research. We then explain how these challenges are further exacerbated in the context of unanchored comparisons.

### 3.1. Small Sample Size and Poor Overlap

#### 3.1.1. Impact on MAIC

One of the key assumptions underlying the MAIC methodology is the positivity of trial assignment, which implies that patients are not deterministically assigned to AgD trial over IPD trial based on their characteristics [9]. Violations of the positivity assumption occur when the eligibility criteria of the IPD trial exclude specific patient subgroups that are present in the AgD trial. Positivity is essential for ensuring sufficient overlap in patient characteristics between the trials and, consequently, the existence of a valid MAIC weighting solution [21].

In small-sample IPD trials, it is likely that certain subgroups present in the AgD trial are not represented in the IPD—not due to explicit inclusion or exclusion criteria, but as a result of sampling variability [22]. In such instances, the positivity assumption is violated purely by chance. This leads to a lack of covariate overlap, and as a result, no valid set of weights can be constructed to match the AgD population.

One might expect that if the range of each covariate in the IPD trial includes the corresponding summary statistics from the comparator trial, then MAIC should be feasible. However, this condition is not sufficient. For a MAIC solution to exist, the aggregated covariate mean vector from the comparator trial (${\bar{X}}_{B}$) must fall within the region covered by the IPD, i.e., the set of all convex combinations of the IPD observations in covariate space [23]. If ${\bar{X}}_{B}$ lies outside this region, no reweighting of the IPD observations can exactly reproduce it. This issue is particularly likely in trials with small sample sizes, where the available IPD may not adequately span the covariate space.

When a solution is attainable despite poor population overlap, the resulting weights often concentrate on a small subset of individuals in the IPD. Notably, and somewhat counterintuitively, these individuals are not necessarily those whose covariates are closest to ${\bar{X}}_{B}$; rather, they tend to lie on the boundaries of the IPD covariate space. This concentration of weights arises from the geometric properties of the MAIC optimization problem. When the target covariate mean vector lies far from the center of the IPD distribution, balancing constraints force the solution toward the boundary of the convex hull of observed covariates, resulting in extreme upweighting of tail observations. Extreme weights create a situation in which the estimated treatment effect is disproportionately influenced by only a few patients, leading to substantial reductions in *ESS* and instability in the estimate [15,21]. The loss of precision and stability are especially pronounced in small samples, where the treatment effect may effectively be driven by the outcomes of only one or two individuals [16].

Small sample sizes and poor covariate overlap are also associated with an increased risk of bias in MAIC [21]. The extent of this bias depends on the type of outcome, with binary outcomes being particularly susceptible—likely due to small-sample bias inherent in weighted logistic regression [24]. Simulation studies have shown that, under such conditions, MAIC can yield more biased estimates than unadjusted indirect comparison methods, such as Bucher’s approach [21].

Standard error estimation is also adversely affected. The performance of sandwich variance estimators depends on several factors, including the outcome type, sample size, and population overlap. The issues are more pronounced for time-to-event outcomes; as shown in a recent simulation study [25], underestimation bias may be observed in scenarios with nominal sample sizes below 150 and low effective sample sizes (*ESS* ≤ 36), indicating poor overlap. For binary out-comes, finite-sample adjustments mitigate some of these issues [26], with bias primarily observed under particularly severe conditions, including low event rates, small sample sizes (*N* = 50), and poor covariate overlap (*ESS* = 12).

The non-parametric bootstrap, although more conservative, can yield unstable estimates in the presence of highly leveraged data and small samples. Moreover, the bootstrap may fail to provide feasible solutions in some iterations. In the aforementioned simulation study, with near-zero event rates, small sample size (*N* = 50), and poor overlap, the bootstrap failure rate reached 29% [25].

#### 3.1.2. Impact on STC

In STC, the existence of a solution to the outcome regression model is generally not dependent on population overlap. An important exception occurs when a categorical covariate includes a level that is present in the aggregate comparator data but entirely absent in the IPD. In such cases, the model cannot estimate the effect for that category. Otherwise, the outcome regression model remains estimable provided that standard conditions for model identifiability—such as the number of covariates not exceeding the number of observations, the absence of perfect multicollinearity or complete separation—are satisfied.

Even when a model is technically estimable, overfitting can lead to unstable and biased predictions. A widely accepted rule of thumb recommends a minimum of ten observations per covariate [27]. For binary and time-to-event outcomes, the “one-in-ten” rule suggests limiting model complexity to one predictive variable per ten observed events [28,29,30]. While these rules do not justify excluding important covariates from the model, as the selection of effect modifiers and prognostic covariates should be guided by subject-matter knowledge, they can serve as indicators of model reliability—particularly in small samples where such thresholds are often difficult to meet.

In cases of poor covariate overlap, the regression model must extrapolate into regions of the covariate space unsupported by the IPD. While STC can theoretically operate under these conditions, this relies on the strong and often unverifiable assumption that covariate–outcome relationships remain valid beyond the observed data. Simulation studies have demonstrated that when this assumption does not hold, bias in treatment effect estimates can occur [21].

The magnitude of bias in STC tends to increase with smaller sample sizes and is further influenced by the type of outcome being modeled [21]. Bias is typically more pronounced for binary outcomes, especially when the data are sparse and key combinations of covariates and outcomes are underrepresented or absent. In simulation studies, considerable bias in odds ratios estimated via logistic regression was observed under conditions of limited sample size and substantial covariate imbalance [14].

Even under correct model specification, STC loses precision as covariate overlap diminishes because the estimand increasingly relies on extrapolation beyond the observed support. Predictions in these regions are high-leverage—made at covariate values far from those used to fit the model—so uncertainty in the estimated coefficients is magnified, yielding larger standard errors.

#### 3.1.3. Comparison of MAIC vs. STC

Comparisons of correctly specified MAIC and STC—particularly for binary outcomes—show that MAIC can exhibit greater bias and loss of precision than regression-based methods [16]. For example, in a simulation with poor overlap and *N* = 200, the average bias for MAIC was −0.144 versus 0.044 for STC; the MAIC bias was considered important [16]. A key advantage of STC is its lower sensitivity to overlap limitations, which allows the inclusion of a larger number of covariates with poor overlap across studies. This makes STC more adaptable in settings where adjustment for numerous prognostic variables and effect modifiers is required—especially relevant for unanchored comparisons in rare diseases.

However, this flexibility comes with a trade-off. MAIC, while generally less efficient, may be more robust to model misspecification [31,32]. Because MAIC does not require an explicit outcome model, it avoids biases that arise from incorrectly specified covariate–outcome relationships. In contrast, STC relies heavily on the correctness of the regression model, and any misspecification can lead to biased estimates—particularly when extrapolating beyond the observed covariate space. Thus, the choice between MAIC and STC involves balancing efficiency and model robustness, with no universally superior method.

The overview of MAIC and STC limitations was presented in Table 2.

### 3.2. Unanchored Evidence

A key challenge of unanchored PAICs arises from the underlying assumption of conditional constancy of absolute effects. This assumption stipulates that, conditional on accounting for all relevant effect modifiers and prognostic variables, the absolute treatment effect remains invariant across different populations. In practical terms, this implies that unanchored comparisons require adjustment not only for all effect modifiers, as in anchored comparisons, but also for all prognostic covariates, including those that may be unknown or unreported. The bias cannot be fully eliminated through PAIC, otherwise. Importantly, we focus here specifically on differences in study populations, as only these can be addressed within PAIC frameworks. Estimates from unanchored comparisons may also be affected by differences in care delivery or clinical context that influence outcomes but are not captured by baseline patient characteristics. In RCTs, such differences are expected to cancel out between treatment arms, unless care delivery acts as an effect modifier. This protection, however, is absent in the single-arm studies that typically underpin unanchored PAICs.

For example, progression-free survival (PFS), a commonly used clinical outcome in oncology, is typically assessed using radiologic imaging to determine disease progression. Because these assessments are conducted at prespecified visits, PFS estimates depend on tumor assessment schedules, including imaging frequency, assessment windows, and the handling of missed assessments [33,34]. A cross-sectional analysis of 163 randomized oncology trials found that less frequent tumor assessment was associated with higher median PFS values [35]. Consequently, apparent differences in PFS may reflect variations in monitoring intensity across trials and care settings rather than true differences in treatment effect. In unanchored PAICs, such outcome-level heterogeneity cannot be addressed through population adjustment and may violate the assumptions required for valid comparison.

Although conditional constancy of absolute effect assumption is considered unlikely to be satisfied in real-world settings, its implications are particularly pronounced in the context of rare diseases. In such settings, disease biology is often poorly characterized and data availability is limited. As a result, key prognostic variables may be unmeasured or entirely unknown, violating the assumptions necessary for unbiased estimation.

Moreover, the need to adjust for a larger number of covariates increases the risk of methodological complications related to small sample sizes and limited covariate overlap between populations. As the number of covariates increases, problems related to the existence of a solution and the stability of the models become more likely.

In the case of MAIC, the inclusion of additional covariates in the weighting model leads to a reduction in *ESS*. This is particularly problematic in small datasets, where the *ESS* may easily fall below 30—a threshold below which MAIC estimates are prone to be biased [36]. On the other hand, it has been shown that in MAIC, omitting prognostic variables from the covariate balancing process introduces greater bias than including them and outweighs the precision loss, even when overlap is poor [37]. As such, arguments based on *ESS* or feasibility concerns should not be used to justify the exclusion of important prognostic variables from the weighting procedure

An advantage of STC compared to MAIC, is its relative insensitivity to covariate overlap issues. This allows for the inclusion of a larger number of covariates in the model, which can help satisfy the assumption of conditional constancy of relative treatment effects.

## 4. Emerging Methodological Refinements to PAICs

The field of PAICs continues to develop as new methodological refinements emerge to address their known limitations and improve their reliability in complex evidence settings. Among these, alternative weighting schemes have been explored to address the limitations of conventional MAIC. For example, Jackson et al. proposed a method that departs from the logistic regression-based propensity score model and instead directly satisfies the method of moments while minimizing the variance of the weights—thus maximizing *ESS* [38]. This optimization approach retains covariate balance but places greater emphasis on weight stability. As such, it may be useful as a sensitivity analysis alongside standard MAIC, or even as a primary analysis in scenarios where maximizing *ESS* is essential to improving estimate precision. However, while this method improves precision, the increase in *ESS* does not guarantee improved performance with respect to bias [38]. Further research is needed to understand the conditions under which this approach may yield more accurate or reliable treatment effect estimates.

Weight truncation is another intuitive enhancement aimed at reducing the influence of extreme weights [39]. However, truncation-based approaches entail an inherent trade-off: while they reduce variance by down-weighting outlier observations, they also alter the target population of inference [40]. This shift occurs because the resulting weighted population no longer corresponds exactly to the comparator population, thereby compromising the internal validity of the adjusted treatment effect estimate. As a result, the gain in precision may come at the expense of bias with respect to the prespecified target.

Bayesian methods are well-suited for small-sample contexts, where frequentist approaches often depend on asymptotic assumptions that may not hold [41]. Unlike frequentist inference, Bayesian statistics combine two sources of information: the prior distribution, reflecting existing knowledge or beliefs, and the likelihood, reflecting the observed data. By combining the two, Bayesian inference yields a posterior distribution from which estimates and credible intervals are derived. The influence of the prior is greater in smaller samples, making its specification particularly important.

In this context, Bayesian STC with G-computation with informative priors has been proposed as a promising approach to stabilize regression estimates when data are sparse [16]. By incorporating external information, this method can reduce variance and improve estimate robustness—particularly relevant for PAIC applications in rare diseases. However, its performance is highly sensitive to the specification of the prior distribution. In rare diseases, where disease mechanisms are poorly understood and comprehensive natural history data are often lacking, defining informative priors can be especially challenging. As a result, while Bayesian methods offer theoretical advantages, their practical utility in rare disease settings hinges on the availability and quality of prior information [42].

The NICE Technical Support Document on PAICs recommends a doubly robust estimation approach, which combines weighting and outcome regression to reduce bias from model misspecification [13]. This involves deriving individual weights—typically via the method of moments in MAIC—and incorporating them into an outcome regression model, similar to STC. The estimator remains consistent if either the weighting or outcome model is correctly specified. Another recent work proposed different augmented estimator for relative effectiveness that combines MAIC weights with an outcome regression to achieve double robustness in unanchored ITCs [32]. Double robustness is obtained by adding to the G-computation estimator an error-correcting term based on MAIC weights rather than relying on predictions from a weighted outcome model. This approach is more generalizable, as for example, it can be applied to comparisons involving time-to-event outcomes. However, the authors noted that the augmented estimator does not eliminate small-sample bias, nor does it relax the fundamental assumption required in unanchored settings (i.e., conditional constancy of absolute effects).

A noteworthy development in the broader landscape of indirect treatment comparisons is Multilevel Network Meta-Regression (ML-NMR), which extends population-adjusted methods to connected evidence networks [43]. ML-NMR can incorporate both aggregate and individual-level data and enables estimation of treatment effects in a decision-relevant target population, rather than being confined to a specific trial population. This flexibility enhances its capacity to address population heterogeneity across studies. However, to date, ML-NMR has only been implemented in anchored settings, requiring a common comparator across trials. This restricts its applicability in rare diseases, where unanchored comparisons are often necessary. Consequently, despite its methodological strengths, the use of ML-NMR in rare disease contexts remains limited.

Another innovative approach for PAIC that extends beyond conventional methods is synthetic data generation (SDG) [44]. This method employs a generative model trained on available IPD to create artificial patient profiles that reproduce real datasets in terms of demographic variables, baseline clinical characteristics, and outcomes. While synthetic datasets were initially developed to facilitate data sharing and protect patient privacy, recent studies have demonstrated their potential for application in the context of indirect treatment comparisons [45,46]. Early evaluations suggest that SDG can yield more precise estimates than MAIC, with gains in precision expected to be particularly valuable in scenarios characteristic of rare diseases, such as limited population overlap [47]. However, despite its promise, synthetic data generation remains methodologically immature for routine HTA use. Validation evidence is still limited, and, to the best of our knowledge, no comprehensive simulation studies have systematically assessed its performance across a range of realistic rare disease settings. As such, SDG should currently be viewed as an experimental extension rather than a substitute for established population-adjusted methods.

## 5. Bias Quantification in PAICs

While the methodological refinements discussed above aim to improve the performance of PAICs under challenging data conditions, they do not eliminate the fundamental sources of uncertainty inherent to PAIC assumptions—most critically, the unverifiable conditions of conditional constancy of relative (anchored) or absolute (unanchored) effects. As a result, improving estimation techniques alone is insufficient to ensure reliable inference. Approaches that explicitly quantify potential bias and assess robustness are therefore also required. The following section focuses on formal bi-as quantification methods developed to evaluate the sensitivity of PAIC-derived estimates beyond conventional measures of statistical precision.

One approach to addressing these challenges is quantitative bias analysis (QBA), which explicitly quantifies the potential impact of systematic errors on treatment effect estimates. This formalization enables investigators to evaluate the robustness of findings and determine the magnitude of bias required to meaningfully alter conclusions.

A promising development in this area is the Extended STC which incorporates QBA techniques from epidemiology into the PAIC framework [48]. Extended STC evaluates the potential impact of unmeasured confounding by simulating the effect of covariates that are not reported in the comparator study. By varying assumptions about these unmeasured variables, the method enables structured sensitivity analyses and provides a more rigorous test of results robustness. Its applicability, however, is limited to covariates absent from the comparator but available in the IPD.

Similarly, the Extension of MAIC employs simulation-based approaches to examine the stability of MAIC results under conditions of extremely poor covariate overlap [49]. This method also allows the re-inclusion of non-overlapping variables in the analysis when adjustment for all covariates is infeasible. The approach is still under development and has not yet achieved wide generalizability.

Importantly, once sensitivity parameters are specified, both Extended STC and Extension of MAIC effectively reduce to their respective standard implementations and therefore inherit the core limitations of STC and MAIC—an issue that is particularly salient in rare disease settings. Moreover, the practical implementation of these methods depends on specific and often demanding informational inputs from external sources (e.g., other studies, registries, or expert elicitation). For Extended STC, sensitivity analyses require specifying the distribution of unmeasured covariates. Interpretation of Extension of MAIC results requires knowledge regarding the plausible magnitude and direction of associations between confounding variables and outcomes.

For anchored PAICs, recent Joint Clinical Assessment (JCA) guidance documents recommend shifted hypothesis testing as an approach to address uncertainty that extends beyond statistical imprecision [50,51]. This approach tests the statistical significance of an estimate against a threshold shifted away from the conventional null hypothesis of no effect. The shifted null hypothesis is rejected only when the whole confidence interval lies on one side of the specified threshold. While theoretically appealing, the practical application of this method is constrained by the lack of clear guidance on how to define shift margins or relate them to the expected magnitude of bias.

The JCA materials also recommend the use of the E-value to assess the potential influence of unmeasured confounders and to evaluate the robustness of the obtained treatment effects. The E-value provides a quantitative measure of the strength of unmeasured confounding that would be required to explain away an observed effect. While conceptually appealing, the interpretability of E-values depends on the availability of credible external information to assess whether confounding of the required magnitude is plausible. In rare disease settings, where knowledge of prognostic factors and their associations with outcomes is often limited, this interpretation is challenging.

Closely related to the E-value the bias factor–adjusted MAIC has been proposed [37]. This “bias factor” combines two components: (1) the maximal possible effect of the unobserved effect modifier on the outcome, and (2) the potential differences in its distribution between trials. The MAIC estimate is subsequently adjusted by this bias factor, yielding a relative effect estimate that accounts for effect modifiers not included in the weighting model. However, the genuine insight provided by this method depends on the credibility of the chosen bias factor. In the absence of strong external evidence, for instance, from high-quality observational studies, the selection of a bias factor becomes a largely arbitrary exercise. In such cases, which are common in many applications, the analysis risks conveying a false sense of rigor by quantifying uncertainty around assumptions that lack empirical foundation and should therefore be applied with extreme caution.

## 6. Discussion

PAICs have become an important component of evidence synthesis in HTAs for orphan drugs. However, as this paper has illustrated, both MAIC and STC are subject to instability, bias, and precision loss when applied under the data constraints typical of rare diseases—namely, small sample sizes, limited covariate overlap, and incomplete knowledge of prognostic factors and effect modifiers. The reliance on unanchored analyses, often unavoidable in rare disease settings, compounds these challenges by introducing strong, unverifiable assumption about the conditional constancy of absolute effects.

In this light, JCA guidance is likely to be particularly important. These documents urge extreme caution in the use of PAICs—not only in rare diseases. According to the guidance materials, anchored approaches may be useful to validate NMA findings when trial similarity is uncertain but are recommended primarily for exploratory rather than primary analyses. The guidance further notes that only very large effect sizes could justify clear-cut decisions based on anchored PAICs. For unanchored settings, it is considerably more restrictive: “only anchored indirect comparisons are appropriate, as these respect within-study randomization”. It also highlights the practical implausibility of the conditional constancy assumption, emphasizing that valid adjustment usually requires access to full IPD from all relevant studies. Finally, as noted, in the small-sample contexts, inclusion of all relevant covariates may be infeasible, rendering population adjustment inappropriate.

We agree that PAICs—especially in rare diseases—have important limitations, and their results should be interpreted with caution. Nonetheless, PAICs are often the only feasible means of generating comparative evidence when head-to-head randomized trials are lacking and IPD from all studies are unavailable, as is frequently the case in rare diseases. Compared with naïve indirect comparisons, PAICs offer a structured and transparent way to reduce bias by aligning trial populations on observed effect modifiers and prognostic covariates. Thus, while PAICs cannot replace randomized evidence, they can provide valuable decision support for HTAs and clinical practice in areas where direct evidence is unattainable.

However, this can be achieved only with additional use of methods that explicitly test robustness. A priority for future research is the further development of formal tools to quantify bias and mitigate the risk of misleading conclusions. Equally important are sensitivity analyses and simulation-based methods that make uncertainty and potential bias more transparent. With such safeguards, unanchored PAICs can provide valuable insights in data-constrained settings, complementing rather than replacing more robust evidence.

To ensure credibility and reproducibility and to mitigate bias, prespecification and transparent reporting should be considered minimum standards for conducting PAIC analyses. This includes prespecifying effect modifiers, prognostic variables, and all key analytical choices (e.g., model selection, handling of missing data) prior to accessing outcome data, in order to reduce the risk of selective covariate selection or data-driven model tuning. Similarly, structured reporting of overlap diagnostics, effective sample size, weight distributions, unadjusted in addition to adjusted treatment effect, and robustness or bias analyses is critical for enabling rigorous appraisal by HTA bodies.

HTA decision-making is a complex, deliberative, and multi-dimensional process in which comparative effectiveness evidence represents only one component among several considerations. Defining the clear-cut conditions under which PAIC-based evidence may inform decision-making is therefore challenging, particularly in rare diseases, as the weight placed on such evidence is inherently context dependent. It may vary according to disease severity, unmet medical need, feasibility of further evidence generation, and the balance between uncertainty in efficacy and evidence on safety. For example, decision-makers may tolerate greater uncertainty in relative efficacy when a treatment has an acceptable safety profile than in contexts where both safety and effectiveness are unclear.

Within this broader framework, PAIC results are most appropriately interpreted as supportive evidence, contributing to HTA deliberations alongside other information sources such as natural history data, real-world evidence, biological plausibility, and external benchmarks.

In practice, while PAICs aim to estimate comparative effectiveness, their application in rare disease settings frequently requires substantial emphasis on the assessment of uncertainty and instability arising from data limitations. Features commonly observed in PAICs—such as substantial reductions in effective sample size, sensitivity of results to covariate selection or modeling assumptions, and reliance on unverifiable assumptions in unanchored settings—provide important signals regarding the robustness of the comparative evidence. Within HTA processes, these considerations may be taken into account in conditional reimbursement decisions, where unresolved uncertainty is explicitly acknowledged. In such contexts, coverage with evidence development may be used to grant patients temporary access to a treatment, conditional on additional evidence generation by the health technology developer, for example through registries or real-world evidence studies. Similarly, diagnostics from PAIC analyses can guide the prioritization and design of further evidence generation by identifying key drivers of instability, such as influential subgroups or covariates. Conversely, in extreme scenarios, where unanchored PAICs combine small sample sizes, severely limited covariate overlap, substantial gaps in information on key prognostic factors and a realistic prospect exists to generate higher-quality evidence, PAIC findings may be most appropriately treated as hypothesis-generating, rather than serving as primary quantitative inputs for reimbursement or pricing decisions.

## Figures and Tables

**Table 1:** Methodological Aspects of MAIC vs. STC.

	MAIC	STC
Core approach	Weights IPD to match aggregate covariates of comparator study; estimates treatment effects in the reweighted IPD	Models outcome as function of covariates using IPD; predicts treatment effect in comparator population
Data requirements	IPD for 1 trial; published aggregate baseline summaries for comparator	
Adjustment mechanism	Weighting of individuals to align covariate distributions	Regression-based modelling of outcomes conditional on covariates
Target population	Comparator population	
Standard error estimation	Sandwich variance estimator or non-parametric bootstrap	Derived from regression model (asymptotic theory or bootstrap)

**Table 2:** Limitations of MAIC vs. STC.

Limitation	MAIC	STC
Positivity/overlap	Requires positivity assumption: sufficient overlap between IPD and comparator populations. Violations occur if comparator subgroups are absent in IPD due to design or sampling variability.	Less sensitive to overlap; model estimable as long as comparator covariate levels are represented in IPD. Extrapolation into unsupported regions increases reliance on unverifiable assumptions.
Small sample size	Extreme weights concentrate on few patients, leading to sharp reductions in *ESS*, unstable estimates, and loss of precision. Small IPD may fail to span comparator covariate space; feasible weighting solution may not exist.	Small samples constrain model complexity. Overfitting leads to unstable predictions. Sparse data exacerbate bias, especially for binary outcomes.
Bias	Bias increases under poor overlap or small *ESS*. Under some conditions, may yield more biased estimates than unadjusted methods (e.g., Bucher).	Bias arises mainly from model misspecification and extrapolation. Logistic regression estimates biased in sparse settings, particularly when key covariate–outcome combinations are missing.
Variance estimation	Sandwich variance estimator underestimates variability in small samples; bootstrap more conservative but unstable when extreme weights or infeasible solutions occur.	Variance estimates generally accurate when model correctly specified.
Relative performance	More robust to model misspecification (no explicit outcome model), but greater sensitivity to overlap issues.	More efficient and less sensitive to overlap, but more vulnerable to model misspecification and extrapolation.

## Data Availability

No new data were generated or analyzed in support of this work.

## Abbreviations

The following abbreviations are used in this manuscript:

AgD	Aggregate-level data
ESS	Effective sample size
HTA	Health technology assessment
IPD	Individual patient data
ITC	Indirect treatment comparison
JCA	Joint Clinical Assessment (EU HTA guidance)
MAIC	Matching-Adjusted Indirect Comparison
ML-NMR	Multilevel Network Meta-Regression
NICE	National Institute for Health and Care Excellence
NMA	Network meta-analysis
PAIC	Population-adjusted indirect comparison
QBA	Quantitative bias analysis
RCT	Randomized controlled trial
SDG	Synthetic data generation
STC	Simulated Treatment Comparison
US FDA	United States Food and Drug Administration
